# A Giant Kidney Stone in a 50-Year-Old Mayan Gardener From the Yucatan Peninsula: A Case Report

**DOI:** 10.7759/cureus.49994

**Published:** 2023-12-05

**Authors:** Ricardo Méndez-Molina, Francisco J Avilés-Murguía, Jose L Millet-Herrera, Diego A Hernández-Castro, Ermilo J Echeverria-Ortegon, Mario Basulto-Martínez, Jessie Langmeyer, Nina Mendez-Dominguez, Juan P Flores-Tapia

**Affiliations:** 1 Clinical Sciences Department, Universidad Marista de Mérida, Mérida, MEX; 2 Nephrology, Urology and Renal Transplant Department, Hospital Regional de Alta Especialidad de la Península de Yucatán, Mérida, MEX; 3 Clinical Sciences Department, Michigan State University College of Osteopathic Medicine, East Lansing, USA; 4 Research and Learning Department, Hospital Regional de Alta Especialidad de la Península de Yucatán, Mérida, MEX

**Keywords:** case report, gardener, mayan population, occupational diseases, staghorn renal stone, urolithiasis

## Abstract

Urolithiasis is a prevalent urological condition characterized by stone formation in the urinary tract, but stones weighing more than 100 g are rare. The Mayan and Mestizo populations in Yucatan have been identified as being at an increased risk of urolithiasis because of the coexistence of environmental, genetic, metabolic, and lifestyle risk factors. The patient’s occupation may play a significant role in enhancing these factors. Here, we report the case of a Mayan gardener with a giant kidney stone weighing 1,154 g, one of the largest ever reported from Mexico.

## Introduction

Just in 2019, 115,552,140 cases of urolithiasis were registered worldwide [1], and therefore, it is considered a common condition. Urolithiasis is stimulated by the interaction of environmental, genetic, metabolic, and lifestyle factors, promoting urine supersaturation. Chronic dehydration due to work environment characteristics and agrochemical exposure can increase the risk of urolithiasis. Occupational aspects, such as working in hot environments or engaging in physically demanding tasks, may contribute to chronic dehydration, which is a risk factor for urolithiasis. Additionally, certain occupations may involve limited access to water or opportunities for regular hydration breaks, further exacerbating the risk [2,3]. This article presents the case of one of the largest ever reported urinary stones in a 50-year-old Mayan gardener from Mexico.

## Case presentation

A 50-year-old male Mayan gardener from a rural community in Yucatan, Mexico, with a significant medical history of hypertension and urolithiasis under medical management, denied a history of drug use. The patient was referred to our tertiary hospital with severe abdominal pain (Visual Analog Scale score of 7/10) associated with hyporexia, left flank tenderness, malaise, constipation, lipothymia, and non-quantified fever; the patient reported urinating hours before admission. Physical examination revealed abdominal distension without audible bowel sounds and a positive left Giordano’s sign. The blood examination results are presented in Table 1. Urinalysis was unremarkable.

**Table 1:** The patient showed anemia, leukocytosis, kidney dysfunction, and electrolyte imbalances.

Test	Unit	Result	Reference range
Hemoglobin	g/dL	9.20	13.00–15.00
Leukocytes	×10^3^/μL	17.19	4.00–12.00
Neutrophils	×10^3^/μL	15.71	2.00–6.00
Creatinine	mg/dL	2.99	0.70–1.20
Urea	mg/dL	93.4	0.00–50.0
Sodium	mmol/L	128	135–145
Potassium	mmol/L	5.3	3.5–5.1
Calcium	mg/dL	7.0	8.4–10.2

**Figure 1:**
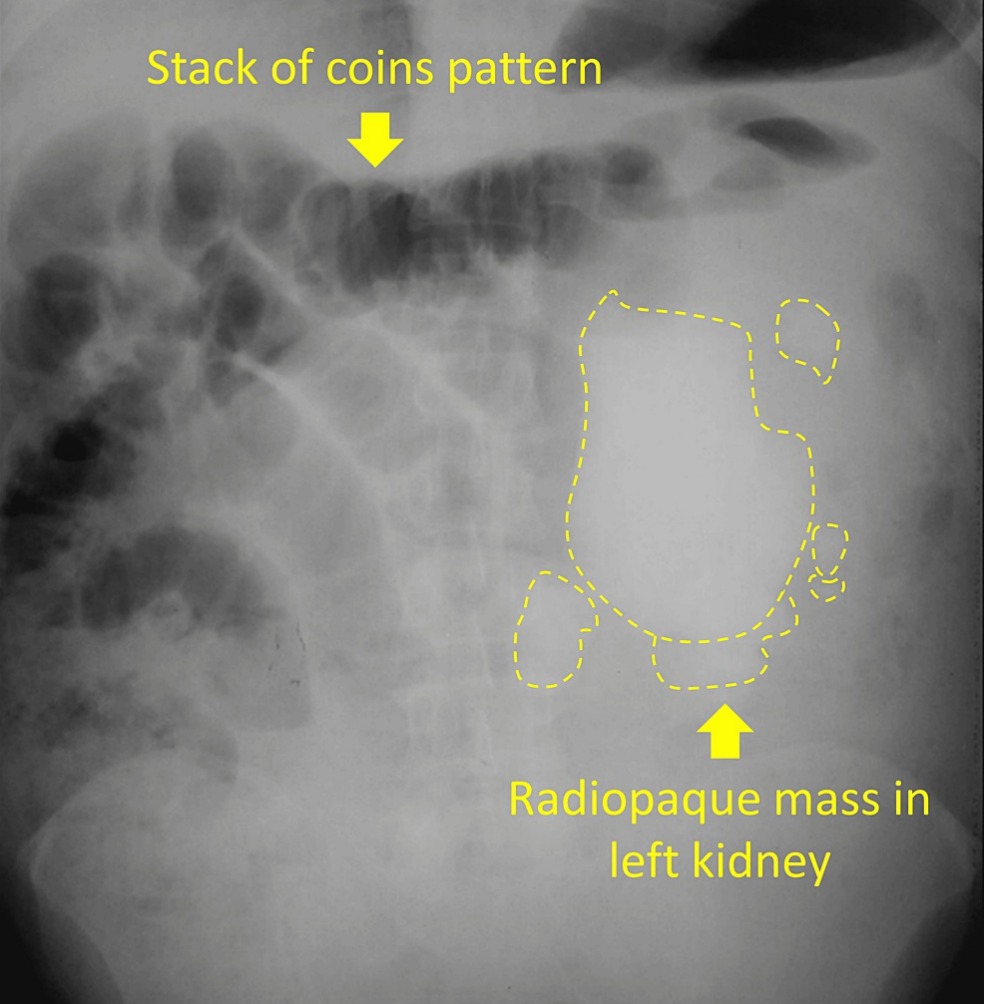
kidney, ureter, and bladder film.

**Figure 2:**
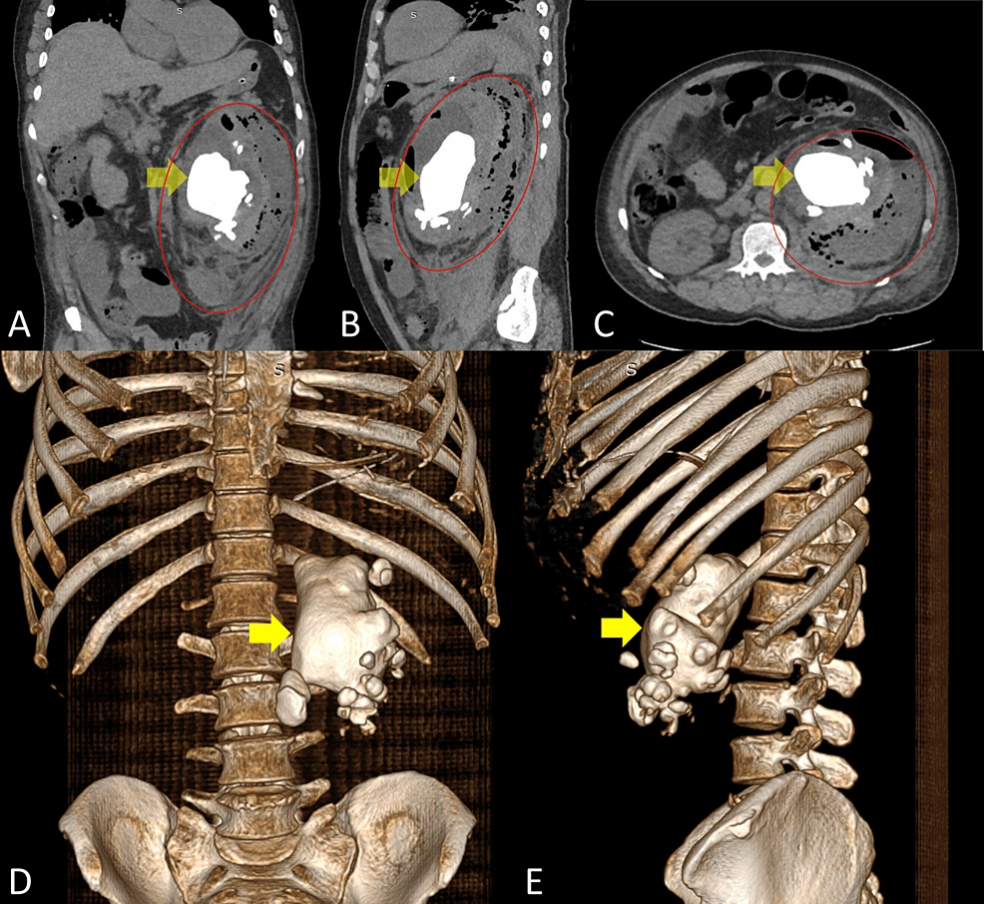
Non-enhanced abdominopelvic CT scan showing staghorn nephrolithiasis with a renal abscess. The yellow arrow points to a hyperdense region indicative of staghorn lithiasis, while the red circle encompasses the hypodense area where the abscess was identified. A: Coronal section. B: Sagittal section. C: Axial section. D: Three-dimensional reconstruction, anterior view. E: Three-dimensional reconstruction, left lateral view.

The kidney, ureter, and bladder film (Figure 1) showed dilated intestinal loops in a stack-of-coins pattern and a radiopaque mass on the left flank consistent with a staghorn kidney stone. A non-enhanced abdominal and pelvic CT scan (Figure 2) revealed a 124 × 64 × 70 mm hyperdense mass in the left kidney involving the entire collecting system and proximal ureter, with a volume of 555 cc and a density of 760 HU. The CT scan showed an associated 179 × 46 × 107 mm heterogeneous, hypodense subcapsular mass extending to the posterior pararenal space, with a volume of 458 cm^3^ and a density of 16 HU.

The patient was urgently shifted to the operating room where open drainage was performed. Moreover, a urinary-guided nephrostomy was performed for further endourologic procedures. Intraoperatively, the patient developed cardiovascular instability, requiring the administration of vasoactive amines. However, due to a poor response, he was transferred to the intensive care unit, where he developed further cardiac arrest. Despite resuscitation maneuvers and advanced cardiopulmonary resuscitation, the patient died six hours later due to septic shock.

## Discussion

We present the case of a 50-year-old male Mayan gardener from Yucatan, Mexico, with a 124 × 64 × 70 mm left kidney stone of 760 HU, a 555 cc computed volume, and a calculated weight of 1,154 g, the largest ever reported from Mexico.

Mayan and Mestizo populations from Yucatan have been identified as being at an increased risk of urolithiasis owing to complex environmental, genetic, metabolic, and lifestyle-related traits [4]. Urolithiasis is endemic to the Yucatan Peninsula region, where hot temperatures and humidity levels may lead to chronic dehydration and subsequent urine supersaturation. The extent of exposure to these environmental factors differs from person to person, with occupation playing a significant role. Field and land workers, especially those from Central America, often work long hours under intense sunlight, leading to high exposure to heat, agrochemicals, and dehydration. These factors have been linked to urolithiasis and chronic kidney disease [5,6].

Gardening is an outdoor economic activity that includes maintaining green spaces and watering, fertilizing, and cultivating plants. Owing to the high temperatures and humidity levels in Yucatan, and journeys that last eight hours on average, gardening can cause consistent dehydration, reduced urinary volume, and, hence, an increase in urine concentration. Several studies have linked high workplace temperatures to urolithiasis [2,3,7]. Additionally, the patient’s vulnerability is an aspect to take into account, as in the patient’s place of residence, gardening is considered casual or contingent work, thus limiting access to employment benefits including health insurance, and delaying the diagnosis until the manifestations became unbearable. Therefore, inadequate precautions could play a significant role in the patient’s profession and contribute to the development of urolithiasis along with the democratization of healthcare.

For drainage, the open approach was considered due to the size of the abscess collection, and guided nephrostomy was performed to prepare for endourologic procedures, which has been proven safe in patients under comparable conditions [8,9]. Some metabolic risk factors have been previously described in the Yucatan population. Mayan patients with stones have a dramatic prevalence of metabolic disorders, such as hypocitraturia (91.3%), hypomagnesuria (68.5%), hypercalciuria (42.1%), hyperuricemia (33.3%), hyperuricosuria (26.6%), and hyperoxaluria (36.5%) [10,11]. Nevertheless, none of these metabolic risk factors were identified in the patient discussed here, which underlines the relevance of occupational, environmental, and lifestyle factors that may play a key role in lithiasis, such as extensive sunlight exposure and dehydration.

The present case highlights the relevance of occupational and environmental factors in urolithiasis development and emphasizes the importance of preventive measures to promote the health of land workers. The report also stresses the need for early evaluation and effective management of patients with urolithiasis to prevent serious complications such as those presented in this case.

## Conclusions

Here, we present one of the largest urinary stones ever reported in Mexico. The patient’s occupation may have played a significant role in terms of other environmental, genetic, and lifestyle determinants that increase urolithiasis development.

Adequate, constant hydration and sunscreen-protected clothing should be recommended by healthcare providers to individuals at occupational risk, including gardeners. Public health measures can lead to comprehensible labeling of fertilizers including key messages targeting health hazards associated with continuous exposure to agrochemicals. Furthermore, greater efforts are needed for the governmental implementation of policies ensuring healthcare access to casual and contingent workers.
